# Primary Outcomes of Patients with Chronic Angle-Closure Glaucoma Treated with Combined Phacoemulsification, Viscogoniosynechialysis, and Endocyclophotocoagulation

**DOI:** 10.1155/2019/6378489

**Published:** 2019-06-13

**Authors:** Juan Carlos Izquierdo Villavicencio, Natalia Agudelo Arbelaez, Bárbara Rubio Lastra, Imelda Ramirez, Fabiola Quezada, Maria Corina Ponte, Laura Cañola, Josefina Mejias Smith

**Affiliations:** Research Department, Oftalmosalud Instituto de Ojos Lima-Perú, Av. Javier Prado Este 1142, San Isidro, Lima, Peru

## Abstract

**Purpose/Objective:**

To evaluate the effectiveness of combined phacoemulsification, viscogoniosynechialysis (VGSL), and endocyclophotocoagulation (ECP) in patients with moderate chronic angle-closure glaucoma (CACG) with peripheral anterior synechia (PAS) ≥90 not controlled with glaucoma medications and previous iridotomy yag laser.

**Materials and Methods:**

We retrospectively reviewed records from patients with cataract and uncontrolled chronic angle-closure glaucoma despite maximal tolerated medical therapy and iridotomy yag laser who received combined treatment with phacoemulsification with posterior capsular lens implantation, VGSL, and ECP 360°. We evaluated intraocular pressure (IOP), glaucoma medications, and best corrected visual acuity (BCVA) preoperatively and during follow-up.

**Results:**

A total of 29 eyes from 22 patients received surgical intervention. Mean follow-up was 6 months. Mean preoperative IOP was 18.2 mmHg, and postoperatively, IOP was 13.5, 12.2, and 12.8 mmHg at 1, 3, and 6 months, respectively. Complete success was 37.9%, and relative success was 72.4%. Mean BCVA was 0.4 logMAR preoperative and 0.3 logMAR 6 months after surgery. Glaucoma medication fell significantly from 2.34 ± 1.66 preoperatively to 1.31 ± 2.6 postoperatively (*p* < 0.001). Overall, 44.8% of the patients did not require glaucoma medications at 6 months. There were no visual significant complications.

**Conclusion:**

Combined treatment with phacoemulsification with posterior capsular lens implantation, VGSL, and ECP is effective and safe in reducing IOP and number of glaucoma medications with stable BCVA at 6 months.

## 1. Introduction

Glaucoma is the second leading cause of irreversible blindness worldwide. By 2020, the increase of the diagnosis of primary glaucoma by 20 million people is expected. While 24% of those with primary glaucoma have CACG, the number of estimated blind is nearly equal to open angle glaucoma because of the greater morbidity of this disease [1]. The spectrum that involves angle closure includes occludable angle, defined as iridotrabecular apposition ≥90° detected on gonioscopy, primary angle closure (PAC), when the mentioned apposition is accompanied with PAS or elevated IOP, and chronic angle-closure glaucoma (CACG) when PAC and optic neuropathy are confirmed with functional and structural tests [2].

First-line treatment of angle closure is iridotomy yag laser with glaucoma medications when IOP is increased [3]. Traditionally, when first-line treatment fails, filtering surgery is indicated with a high rate of complications described such as shallow anterior chamber, hypotony, choroidal detachment, choroidal hemorrhage, and aqueous misdirection with significant visual impairment as a result [4].

Other treatments that could be used before filtering surgery have been investigated including phacoemulsification alone or combined phacoemulsification with other procedures [5]. Phacoemulsification has shown to effectively reduce IOP in angle-closure glaucoma by deepening the angle due to increase in anterior chamber width with less complications and better visual outcomes. Chen et al. reported an IOP reduction of 30% in CACG and 71% in acute PAC with a significant reduction in glaucoma medications [6].

Several studies had described the outcomes of the combination of phacoemulsification and VGSL with the aim to open the angle and restore mechanical obstructed trabecular meshwork drainage. Harasymowycz et al. found a 62% reduction of mean IOP and 55% reduction of glaucoma medications with this combined treatment for the management of unresponsive primary angle-closure glaucoma [7, 8].

ECP was first suggested by Thorpe in 1934 and developed by Uram in 1992. Its effectiveness and safety concerns to lower IOP have been demonstrated [9]. It uses an endolaser diode energy probe with an endoscope that directly visualizes and precisely photocoagulate ciliary processes, with the aim to reduce production of aqueous humor. When combined with phacoemulsification, it can reduce glaucoma medications at 4 to 6 weeks after the procedure when maximal effect is observed [10–13].

We aimed to evaluate the effectiveness and safety of the mentioned procedures for reducing IOP, first by opening the drainage angle with combined phacoemulsification and VGSL and then reducing aqueous humor production with ECP in patients with CACG.

## 2. Methods

We retrospectively reviewed the medical records from patients with moderate CACG with PAS ≥90° not controlled with glaucoma medications and iridotomy yag laser with significant visual impairment from cataract who underwent combined phacoemulsification with capsular bag intraocular lens implantation, VGSL, and ECP at the Institute of Eyes Oftalmosalud, Lima, Peru, between August 2017 and July 2018. The study protocol complied with the principles of the Declaration of Helsinki, and it was reviewed and approved by the Ethics Committee of the Institution. An informed consent was obtained from every patient.

Inclusion criteria were as follows: patients older than 50 years old, diagnosis of moderate chronic angle-closure glaucoma (moderate defined with visual field DM −6 to −12 dB and retinal nerve fiber layer in OCT NIDEK <80 and >70 microns) with progression (confirmed with 2 visual fields) despite previous iridotomy and glaucoma medications, and cataract with BCVA less than 20/40; exclusion criteria were as follows: follow-up less than 6 months, other intraocular surgeries including selective laser trabeculoplasty (SLT) and retinal or corneal surgeries, and patients who had complications during phacoemulsification.

Preoperatively, all patients underwent complete ophthalmic evaluation including visual acuity (VA) by Snellen testing, slit-lamp biomicroscopy anterior and posterior evaluation, and IOP measurement with a Goldmann applanation tonometer. The number of glaucoma medications used was recorded. Patients were reviewed postoperatively at day one, after one week, and at 1, 3, and 6 months. Complete success was defined as an IOP ≤14 mmHg without the need for glaucoma medications. Relative success was defined as an IOP ≤14 mmHg with or without glaucoma medications.

**Figure 1:**
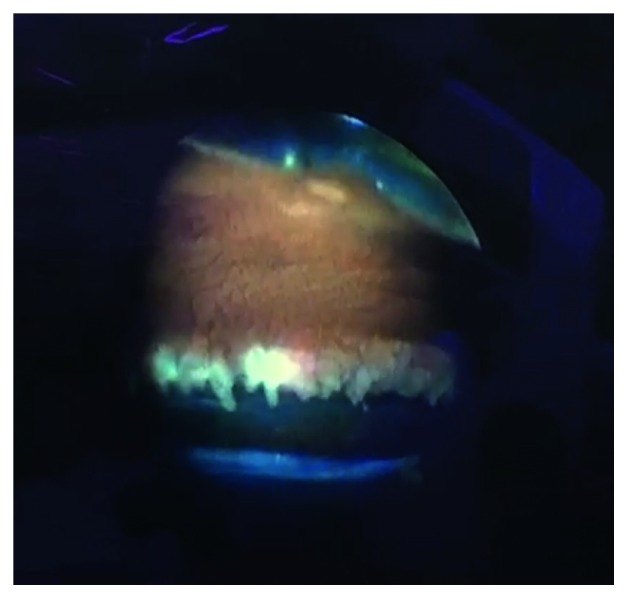
Ciliary processes whitening visualization with an endoscope.

Experienced glaucoma specialist or glaucoma fellow performed the standard phacoemulsification using the CENTURION vision system (Alcon, Texas, USA) and posterior capsular lens implantation in all cases. Then, ECP was performed using E2 Laser and Endoscopy System (Endo Optiks®, New Jersey, USA); after viscosurgical device expanded ciliary sulcus, a 20-gauge probe is advanced to visualize ciliary processes (Figure 1). Three-hundred sixty degrees (360°) treatment was performed with continuous diode laser 810 nm wavelength delivery at 0.20 watt, until whitening and shrinkage occur, avoiding explosion of the tissue. VGSL was performed last using a nontouch procedure to break PAS with Healon® GV (Abbott Medical Optics, Haryana, India) directed to the angle for 360°; direct visualization with the endoscope system was used to confirm 360° angle opening; in some cases, Healon GV was used twice. All patients received intracameral diluted triamcinolone 50 mg/5 ml and 0.1 ml of cefuroxime 50 mg/5 ml.

Postoperative treatment was done with tobramycin 3 mg/ml and dexamethasone 1 mg/ml drops every 4 hours for 7 days, atropine 1 mg drops every 12 hours for 7 days, and nepafenac suspension 1 mg/ml every 8 hours for 15 days.

### 2.1. Statistical Analysis

Data were analyzed with SPSS software ver. 21, using the Friedman test to analyze IOP, number of glaucoma medications, and BCVA results for global significance and chi-square. A *p* value < 0.05 was considered to indicate a significant difference before and after the procedure.

## 3. Results

A total of 29 eyes from 22 patients received combined therapy with combined phacoemulsification, VGSL, and ECP. Patient demographic data are described in Table 1. Mean age was 66.59 ± 10.64; there were 6 male patients (27.3%) and 16 females (72.7%), and 13 were right eye (44.8%) and 16 were left (55.2%). Mean follow-up after phacoemulsification, ECP, and VGSL was 6 ± 1.4 months.

### 3.1. Intraocular Pressure

**Figure 2:**
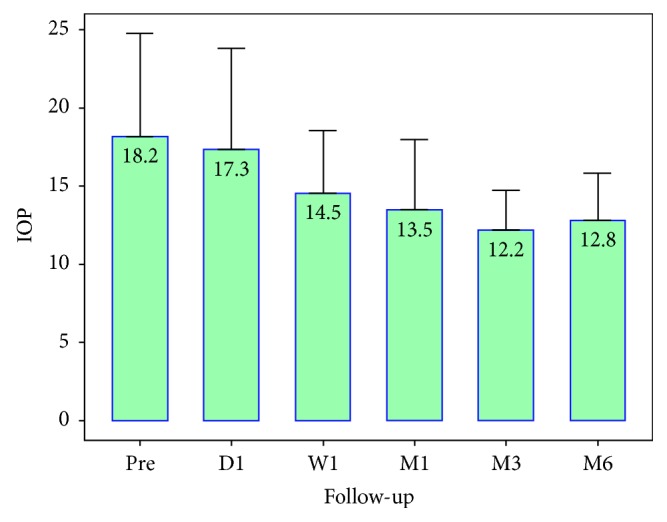
Change in IOP preoperatively and postoperatively.

Mean preoperative IOP was 18.2 ± 6.6 mmHg; mean postoperative IOP was 13.5 ± 4.5 mmHg, 12.2 ± 2.5 mmHg, 12.8 ± 3.0 mmHg at 1, 3, and 6 months, respectively (Figure 2). There were 10/29 (37.9%) patients that had complete success, and 19/29 (72.4%) had relative success.

### 3.2. Visual Acuity

**Figure 3:**
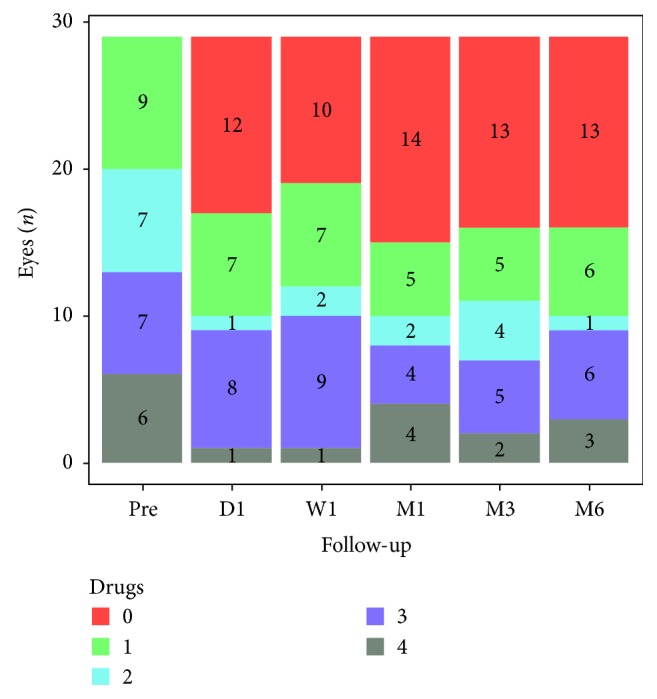
Change in glaucoma medications preoperatively and postoperatively.

**Figure 4:**
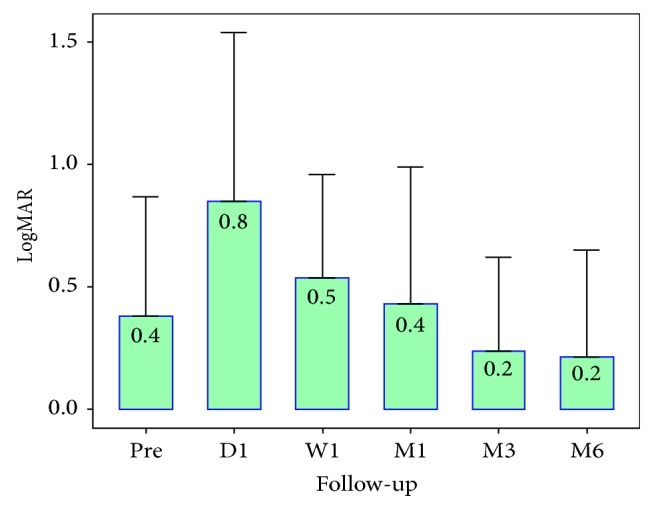
Change in visual acuity logMAR preoperatively and postoperatively.

Mean BCVA was 0.4 logMAR preoperative and 0.8, 0.5, 0.4, 0.2, and 0.3 logMAR, at first day, 1 week, 1 month, 3 months, and 6 months after surgery, respectively (Figures 3 and 4).

### 3.3. Glaucoma Medication

Preoperatively, 9 (31%) eyes had one glaucoma medication, 7 (24.1%) eyes had 2, 7 (24.1%) had 3, and 6 (20.7%) eyes had 4 glaucoma medications. At 6 months postoperatively, 13 (44.8%) eyes did not need any glaucoma medication, 6 (20.7%) were with 1, 6 (20.7%) with 3, 1 (3.4%) with 2, and 3 (10.3%) eyes with 4 glaucoma medications. Glaucoma medication fell significantly from 2.34 ± 1.66 preoperatively to 1.31 ± 2.6 postoperatively (*p* < 0.001).

### 3.4. Complications

There were no significant intraoperative complications. There were 2 complications, one patient with posterior capsular opacification that was treated successfully with yag laser capsulotomy at first month and the second patient had a persistent dilated pupil (Urretz-Zabalia syndrome) that was treated with pupilloplasty.

## 4. Discussion

ECP is a complementary treatment to phacoemulsification and VGSL in patients with CACG and cataract to reduce aqueous humor production to control IOP [9, 14]. It has an acceptable safety profile compared with traditionally filtering surgery, and it could be considered a second-line treatment when iridotomy and glaucoma medications fail to control IOP [9]. In this study, we found a significant reduction in IOP and glaucoma medications; 44.8% patients did not need any glaucoma medication at 6 months after surgery.

Plateau iris syndrome (PIS) is one of the known causes for angle closure; abnormal anterior position of ciliary process may narrow the angle despite peripheral iridotomy. ECP has been described as a possible treatment for this pathology [15], by causing shrinkage of ciliary processes occur, therefore opening the angle. Hollander et al. reported a series of 9 patients with PIS in whom ultrasound biomicroscopy was done before and after cataract extraction and ECP; they reported successful opening where ECP was applied and remained occludable in untreated areas [11]. A prospective study from Mansoori et al. found a prevalence of 32% of PIS in CACG with UBM [15]. Our results showed a significant reduction of mean IOP and glaucoma medications that could be explained due to flattening of the ciliary processes 360° that cause to open the angle 360°.

Razeghinejad et al. found in refractory acute angle-closure glaucoma patients (PAS 270° or less) unresponsive to iridotomy and medical therapy with a mean preoperative IOP of 39.4 mmHg and mean number of glaucoma medications 3.8 treated with combined phacoemulsification and VGSL, mean IOP postoperatively decreases to 13.4 mmHg with mean 0.4 glaucoma medication [8]. In chronic angle-closure glaucoma, there may be trabecular irreversible dysfunction and open drainage angle but not functional drainage angle [16]; related to our study, we found less reduction in IOP and glaucoma medications, with a mean preoperative IOP of 18.2 mmHg and mean postoperative IOP 12.8 mmHg at 6 months, and reduction of glaucoma medications which is statistically significant; it is important early treatment in angle-closure glaucoma before irreversible damage of the trabecular meshwork is established, close follow-up of those without complete response to iridotomy is mandatory, and phacoemulsification with combined procedures like VGSL and ECP should be done before 6 months.

White et al. found, in chronic angle-closure glaucoma treated with phacoemulsification and goniosynechialysis, reduction of IOP preoperatively from 19.8 ± 4.4 mmHg to postoperative 14.4 ± 2.1 mmHg after 6 years of follow-up [16]. Similar to our results, our final IOP postoperatively was 12.8 mmHg at 6 months; larger follow-up is needed to determine reduce control over time, and addition of ECP helps to reduce final IOP and a significant reduction in glaucoma medications.

In a study published by Morales et al. in patients with advanced PACG treated with phacoemulsification and ECP, they report an overall success rate of 13.6% and qualified success of 42% and their target IOP was <15 mmHg; difference might be the use of viscogoniosinechialysis to restore trabecular meshwork drainage in our study that helps treating the primary mechanism involved in this pathology; opening the angle with a viscosurgical device is easy and quick to perform contributing to improve drainage through the conventional pathway and may be the explanation of the best results found in the present study [10].

Phacoemulsification as a solo procedure leads to a decrease in intraocular pressure even if the patient does not have glaucoma; this decrease may be greater in patients with angle-closure disease. Chen et al. in their report by the American Academy of Ophthalmology of the effect of phacoemulsification on intraocular pressure in glaucoma patients found, in 9 studies with a total of 461 patients and mean follow-up of 17 months, a reduction of 13% in primary open angle glaucoma; for pseudoexfoliation glaucoma, in 5 studies with 132 patients and mean follow-up 34 months, 20% reduction; for chronic PACG, in 12 studies with a total of 495 patients and follow-up 16 months, 30% reduction of IOP; and for acute PACG, in 4 studies with 119 patients, and 24 months follow–up, 71% reduction from presenting IOP [6]. Armstrong et al. found in their meta-analysis an effect of at least of 36 months with gradual loss of the initial effect noted after 2 years; the present study found follow-up reduction of IOP with a combined treatment at 6 months [17].

Various techniques have been described to break the peripheral anterior synechiae including Nd : YAG laser and drysdale spatula using swan-jacobs lens [8]. In this study, we use a “no touch” technique easy to perform, in the same phacoemulsification procedure, and confirming angle opening with direct endoscopy visualization; as described by Fang et al., with this technique, no tilt of the operating microscope is need, the patients head remain upright during surgery, it is less invasive, it allows quality images of angle structures, and manipulation is more accurate [14].

To our knowledge, there is only one paper by Alaghband et al. that reports this combination treatment in 3 patients, called PIECES (phacoemulsification with intraocular implantation of lens, ECP and endoscopic goniosinechialysis). Their report includes 3 patients with CACG and PAS ≥270°; the IOP was preoperatively 26, 45, and 40 mmHg, and postoperative reduction of IOP was 18, 10, and 10, respectively, at 12 months. In our study, mean preoperative IOP was 18.2 ± 6.6 and at 6 months, postoperative IOP was 12.8 ± 3.0 [9].

The main weakness of this study was the retrospective nature, short follow-up, and no control group. Our main outcome was to evaluate IOP change after treatment, and it was a retrospective study where UBM was not performed routinely in this patients. It is important in future studies an UBM previous to ECP treatment in CACG to determine the ciliary process anatomic position to differentiate plateau iris syndrome.

In conclusion, the clinical implication of this study is that combined treatment with phacoemulsification with posterior capsular lens implantation, VGSL, and ECP may be effective and safety in reducing IOP and number of glaucoma medications. A prospective randomized clinical trial is required to compare ECP with the gold standard filtration surgery.

## Figures and Tables

**Table 1:** Clinical and demographic characteristics of patients with CACG.

	*N* (%)
No. of patients	22 (100)
Mean age (years)	66.59 ± 10.64
No. of eyes	29 (100)
Right	13 (44.8)
Left	16 (55.2)
Sex	
Female	16 (72.7)
Male	6 (27.3)
Previous iridotomy yag laser	29 (100)
Baseline glaucoma medications	2.34 ± 1.66
Baseline IOP (mmHg)	18.2 ± 6.6
Visual field mean basal DM (db)	−4.5 ± 1.5
Mean RNFL (microns)	75 ± 3.8

## Data Availability

The data used to support the findings of this study are available from the corresponding author upon request.
